# Visualization Methods for DNA Sequences: A Review and Prospects

**DOI:** 10.3390/biom14111447

**Published:** 2024-11-14

**Authors:** Tan Li, Mengshan Li, Yan Wu, Yelin Li

**Affiliations:** 1 School of Physics and Electronic Information, Gannan Normal University, Ganzhou 341000, China; litan@gnnu.edu.cn (T.L.); 1230880004@gnnu.edu.cn (Y.L.); 2 School of Mathematics and Computer Science, Gannan Normal University, Ganzhou 341000, China; 0700101@gnnu.edu.cn

**Keywords:** computational biology, visualization method, graphical representation, knowledge graph, machine learning

## Abstract

The efficient analysis and interpretation of biological sequence data remain major challenges in bioinformatics. Graphical representation, as an emerging and effective visualization technique, offers a more intuitive method for analyzing DNA sequences. However, many visualization approaches are dispersed across research databases, requiring urgent organization, integration, and analysis. Additionally, no single visualization method excels in all aspects. To advance these methods, knowledge graphs and advanced machine learning techniques have become key areas of exploration. This paper reviews the current 2D and 3D DNA sequence visualization methods and proposes a new research direction focused on constructing knowledge graphs for biological sequence visualization, explaining the relevant theories, techniques, and models involved. Additionally, we summarize machine learning techniques applicable to sequence visualization, such as graph embedding methods and the use of convolutional neural networks (CNNs) for processing graphical representations. These machine learning techniques and knowledge graphs aim to provide valuable insights into computational biology, bioinformatics, genomic computing, and evolutionary analysis. The study serves as an important reference for improving intelligent search systems, enriching knowledge bases, and enhancing query systems related to biological sequence visualization, offering a comprehensive framework for future research.

## 1. Introduction

With the rapid advancements in high-throughput sequencing and genomic technologies, the acquisition of massive amounts of biological data has become possible. The pressing concern is how to efficiently extract and interpret this massive amount of data [1,2,3,4]. Bioinformatics integrates theories and methods from various fields, such as computer science, mathematics, and information science. This integration enables effective analysis of biological data [5,6]. Graphical representations provide intuitive information for analyzing biological sequences. Such alignment-free methods exhibit notable features, such as clarity, concise mathematical description, and low time complexity [7,8,9]. Visualization methods have widespread applications across various research domains, including similarity analysis, functional region identification, and species evolutionary relationships. Moreover, the visualization methods of RNA and protein sequences are often based on DNA sequences. Therefore, the development of good DNA sequence visualization methods remains a need in contemporary bioinformatics [10].

Research related to the visualization of DNA sequences lacks a comprehensive classification and summary of methods. It tends to summarize and analyze only a few representative or distinctive methods. Furthermore, the current research databases contain a plethora of visualization methods but lack tools for logically collecting, organizing, and establishing relationships between these methods. A knowledge graph is an effective method for integrating and analyzing large datasets, enabling the organized management of vast amounts of information. Additionally, advanced machine learning methods hold the potential for application in the field of biological sequence visualization.

## 2. Two-Dimensional Visualization of DNA Sequences

### 2.1. Dynamic Walking Model

**Figure 1:**
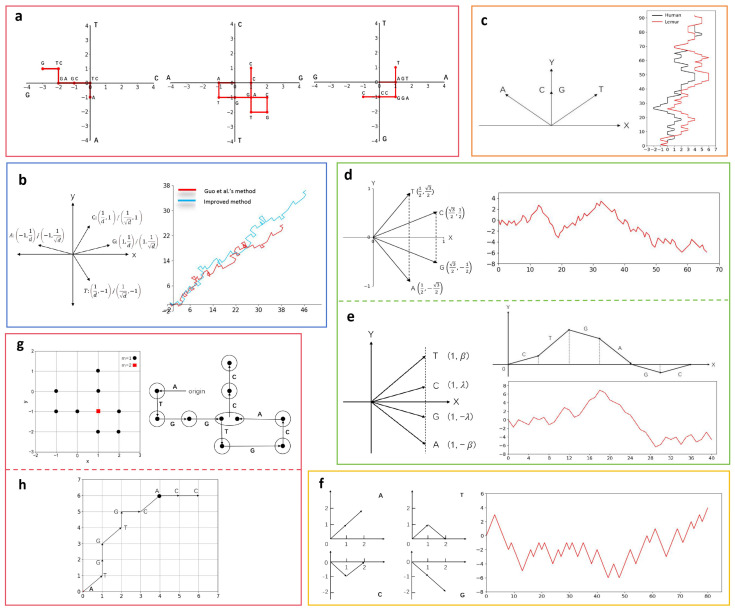
Two-dimensional visualization methods based on the dynamic walking model and the corresponding graphical representations (the sequences used are the first exon of the *human*, *lemur*, and *opossum β-globin* genes). (**a**) Method of Gates et al. [12], Nandy et al. [13], and Leong et al. [14] (**b**) Method of Guo et al. [15] and a further improved method (*d* = 2). (**c**) AT DB curve (Dual-Base Curve). (**d**) Method of Yua et al. [16]. (**e**) H–L curve. (**f**) DV curve (Dual-Vector Curve). (**g**) Method based on point weights. The points with a weight of 2 are represented by red squares, while the points with a weight of 1 are represented by black circles. (**h**) Method based on binary encoding. The circle at A indicates that a degradation phenomenon may occur at this location.

The dynamic walking model involves mapping the original sequence onto a curve on a plane using distinct two-dimensional vectors to represent the four bases A, T, C, and G. The specific steps are as follows: starting from the origin, a series of vectors is generated by sequentially examining the bases in the DNA sequence. For each examined base, the corresponding vector is moved from the current point to obtain the coordinates of the point corresponding to each base. Connecting all the points obtained results in the curve [11]. The two-dimensional visualization methods introduced in this section are shown in Figure 1.

The initial two-dimensional visualization method based on a dynamic walking model was proposed by Gates et al. [12]. Gates et al. used this method to compare the divergent regions of exonic sequences in two *actin* genes. They proposed that the general characteristics of sequence categories could be examined by calculating the fractal dimension of each sequence within this space. Subsequently, a series of similar methods were proposed, such as the ones by Nandy et al. [13] and Leong et al. [14] (Figure 1a). These methods provide different perspectives for intuitively revealing information within DNA sequences. However, these methods may potentially encounter degeneracy and information loss issues. The degeneration problem refers to situations where the process of generating a visual representation results in overlaps and self-intersections, leading to loops or cycles in the final graphic. These issues cause a loss of one-to-one correspondence between the sequence and the two-dimensional curve, meaning that different sequences may share the same graphical representation. As a result, observers cannot uniquely reconstruct the original DNA sequence from a graphic with degeneration problems, leading to a loss of information from the sequence to the graphical representation. Guo et al. improved the coordinate vectors of Nandy et al.’s method by adjusting the vectors to form a small angle with the coordinate axes. They introduced a slight positive deviation of *1/d* along the original axis direction, which reduced the probability of degeneration. Subsequently, other researchers further refined Guo et al.’s method [15] (Figure 1b). This approach has been applied in DNA sequence comparison and similarity analysis by Guo et al.

To mitigate degeneracy and effectively represent DNA sequence characteristics, the DB curve (Dual-Base Curve) uses three vectors to represent the four bases, and two of bases are assigned to the same vector with the y-component set to 1 [17]. The DB curve represents the properties of the two bases. For example, the AT DB curve refers to a vector assignment method where the bases C and G are represented by the same vector (0, 1), while A and T are represented by the vectors (−1, 1) and (1, 1), respectively. This approach places greater emphasis on the relationship between bases A and T (Figure 1c). The DB curve monotonically increases along the positive y-axis without degeneracy. However, because two bases are assigned the same vector (0, 1), it becomes impossible to distinguish which base corresponds to the upward-extending segments of the curve, inevitably resulting in substantial information loss. The local extrema on the DB curve can represent local variations in the relative abundance of the two bases, allowing for a direct visual assessment of their relative abundance. Subsequently, Yau et al. made further improvements to the vector design [16]. The resulting curve appears only in the first or fourth quadrant and exhibits a monotonic increase (Figure 1d). Following the ideas of Yau et al.’s method, subsequent researchers have proposed similar approaches [18,19]. For example, the H–L curve sets the x-component of each vector to 1 in the vector design [20]. The projection of the curve onto the x-axis corresponds to the position of the nucleotides (Figure 1e). Based on the presented H-L curve, Huang et al. proposed a method for determining the type, location, and extent of DNA mutations. This provides an intuitive and simple approach to the rapid diagnosis of genetic diseases. The DV curve (Dual-Vector Curve) addresses the difficulties of observing lengthy DNA sequences in a two-dimensional space [21] (Figure 1f). The DV curve uses two vectors to represent each base and exhibits favorable symmetry. It can be applied to mutation analysis, where relevant DV curves can be examined to quickly identify the location of gene mutations and immediately determine the type of mutation. Additionally, it can be used for similarity analysis.

In addition, some researchers have proposed improved methods based on the Gates method. For example, a method considers the DNA sequence as a set of points with varying weights on a coordinate plane [22]. The weight of a point corresponds to the number of times it is traversed. To prevent degeneracy, different symbols are assigned based on the weight of each point [23,24] (Figure 1g). The method completes a set of descriptors by orienting the graph in a two-dimensional space. Then, it defines a similarity measure based on the comparison of graph positions. Another method involves converting the sequence into a binary encoding [25]. Degeneracy is avoided by using movement rules and distinct symbols to represent the points (Figure 1h). This method, based on the encoding model of artificial metabolic systems, can be used to compare different DNA sequences. Hu et al. studied the similarity of DNA sequences from various animals by analyzing metabolic intermediates.

### 2.2. Spectral Visualization Model

**Figure 2:**
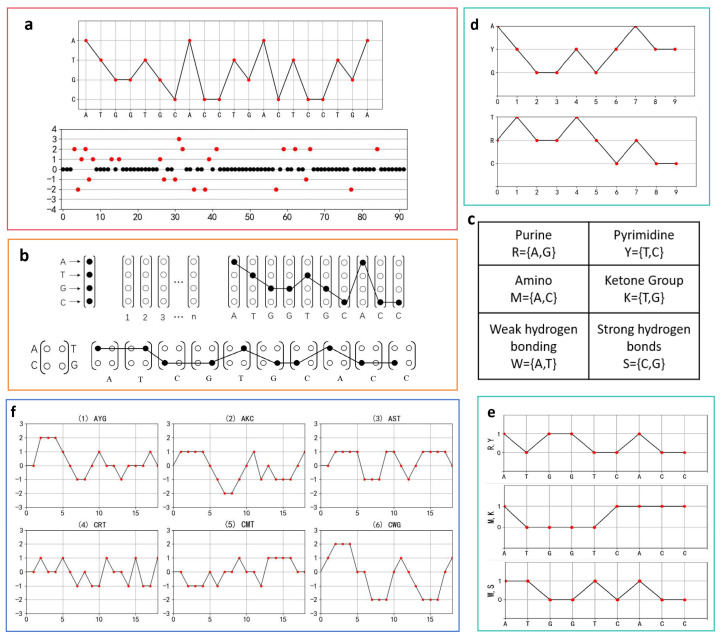
Two-dimensional visualization methods based on spectral visualization models and the corresponding graphical representations (the sequences used are the first exon of the *human* and *lemur β-globin* genes). (**a**) Method of Randic et al. [28] (**b**) Methods based on graph cells. (**c**) Three characteristic classes of nucleotides. (**d**) Method based on the three categories of nucleotide to construct the three horizontal lines. (**e**) Method based on binary form and the three categories of nucleotide. (**f**) Method based on six characteristic classes.

A nondegenerate two-dimensional visualization method maps four bases onto four horizontal lines at a distance of one unit [26,27]. This type of graphical representation resembles a spectral wavy curve that extends horizontally and is restricted to a limited range of spectra in the vertical direction. This is called the spectral visualization model and is illustrated in Figure 2.

This type of method was first proposed by Randic et al. The graphical representations obtained by this method avoid issues of degeneracy and information loss, offer simplicity and intuitiveness, facilitate sequence comparison, and display the sequence length and nucleotide content (Figure 2a). However, when the sequence length exceeds 300 base pairs (bp), the curve becomes very dense, reducing visualization clarity. Using this approach, Randic et al. analyzed the similarities and differences in the first exon coding sequences of the *β-globin* gene across different species. Zhang et al. used this method to analyze the similarity of the *β-globin* genes across seven different species [28]. Then, the concept of a graph cell is proposed [29] (Figure 2b). Designing graph cell units in various forms results in different models [30]. Building on this method, Yao et al. proposed a new similarity analysis technique to examine the similarities and differences among the first exon coding sequences of the *β-globin* gene across various species.

However, the methods discussed above do not account for the physicochemical properties of nucleotides in the sequence. Therefore, some researchers have proposed methods that combine the physicochemical properties of nucleotides. Three horizontal lines are constructed using the three characteristic categories of nucleotides (Figure 2c), yielding the purine and pyrimidine graphical representations [31] (Figure 2d). Song et al. demonstrated its practicality on the first exon coding sequence of the *human β-globin* gene. Alternatively, the sequence can be transformed into binary form: 1 represents nucleotides of the R, M, and W types, while 0 corresponds to nucleotides of the Y, K, and S types. This method produces three 01 feature sequences corresponding to the R–Y, M–K, and W–S classes [32] (Figure 2e). Liao et al. also applied this method to assess the similarities and differences in the first exon coding sequences of the *β-globin* gene across different species. These three characteristic categories can be further divided into six classes (Figure 2f), which correspond to six distinct graphical representations.

### 2.3. Nucleotide Combination Visualization Models

**Figure 3:**
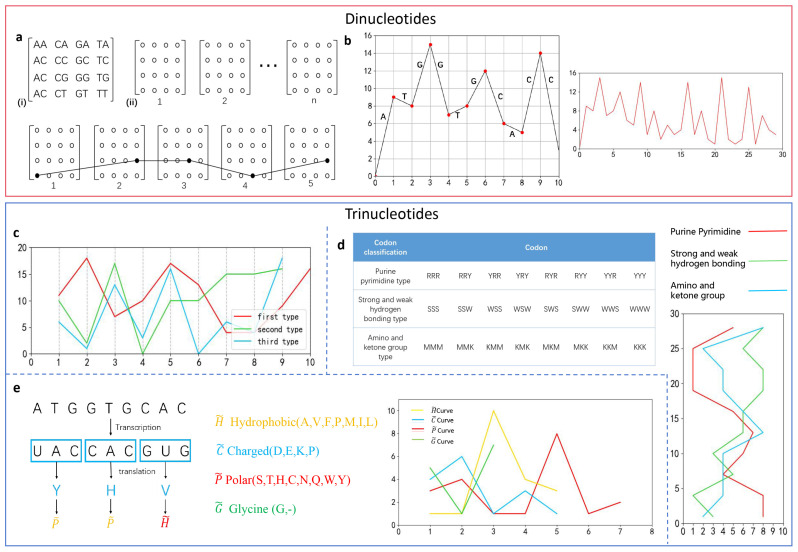
Two-dimensional visualization methods based on nucleotide combination and the corresponding graphical representations (the sequence used is the first exon of the *human β-globin* genes). (**a**) PNN curve. (i) A cell unit contains 16 adjacent nucleotides. (ii) A system composed of cell units. (**b**) DN curve (Dual-Nucleotides Curve). (**c**) Codon-based mapping method. (**d**) Method based on codon classification and classification table of triplet codons. (**e**) Method based on amino acid classification and the translation process according to the first reading frame.

Two-dimensional visualization methods based on nucleotide combination simultaneously consider the nucleotide composition and physicochemical properties of a sequence. Compared to visualization methods based on a single nucleotide, this approach encompasses more biological information. This reduces the computational burden of the alignment and makes it more suitable for handling long sequences, as shown in Figure 3.

For dinucleotides, there are PNN curves based on adjacent nucleotides [33]. The abbreviation “PNN” stands for “pair of the neighboring nucleotides”. These curves design graph cell units, with each cell unit encompassing 16 adjacent nucleotides arranged according to their physicochemical properties (Figure 3a). Xiao et al. used PNN curves to examine the similarities and differences in the first exon coding sequences of the *β-globin* gene across 11 different species, obtaining a distribution of PNNs and new invariants for digital characterization of primary DNA sequences. The DN curve (Dual-Nucleotides Curve) directly represents adjacent nucleotides as vectors [34]. By using a sliding window to read dinucleotides along the sequence, adjacent nucleotides are mapped as a series of points (Figure 3b). The DN curve provides information about the frequency distribution of dinucleotides in given sequences. Qi et al. applied the DN curve to relate each sequence to the mutational characteristics of adjacent bases, generating a seven-component vector relative to seven different mutation types to characterize and compare the first exon coding sequences of the *β-globin* gene across 11 species.

For trinucleotides, DNA sequences are divided into a series of codons and establish mappings [35]. As there are three reading frames, three curves are obtained (Figure 3c). Codons can also be categorized into three groups according to the physicochemical properties of the nucleotides [36]. Three curves are obtained using three types of mapping (Figure 3d). According to the genetic code table, DNA sequences can be translated into amino acid sequences based on reading frames. Based on the physicochemical properties of amino acids, 20 amino acids are classified into four groups. The DNA sequence is converted into a sequence comprising these four characteristics [37] (Figure 3e). The above methods have also been used by researchers to examine the similarities and differences in the first exon coding sequences of the *β-globin* gene across 11 different species.

### 2.4. Other Special Models

**Figure 4:**
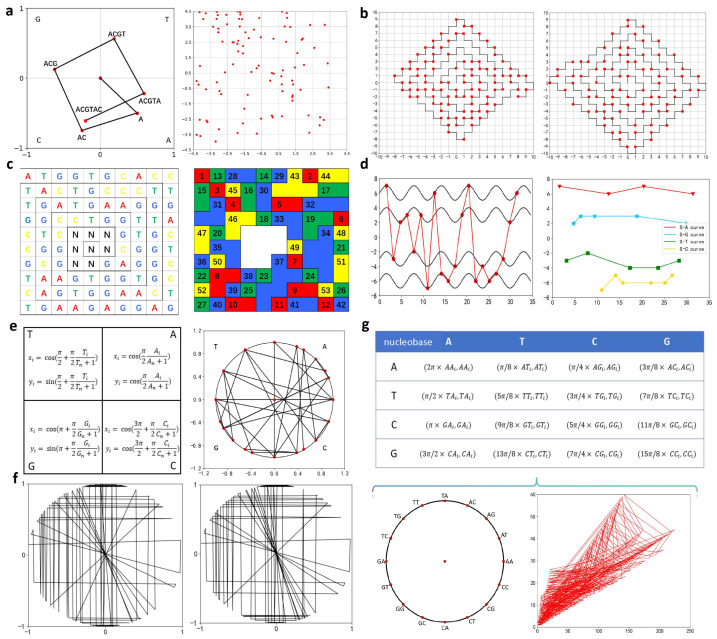
Two-dimensional visualization methods based on special models and the corresponding graphical representations (the sequence used is the first exon of the *human* and *goat β-globin* genes). (**a**) AG-CGR and CGR scatter plots. (**b**) WormBin and WormStep. (**c**) Color5 visualization model. (**d**) The red curve in subfigure on the left represents S-F-B curve (sinusoidal four-base related curves), while the curves in subfigure on the right represent the S-A, S-G, S-T, and S-C curves. (**e**) B-curve. (**f**) DUC curve. (**g**) The method positioned the 16 pairs of adjacent nucleotides on the unit circle, coordinate mapping table of dinucleotide, and circular distribution of adjacent nucleotides.

The two-dimensional methods based on other special models are shown in Figure 4.

The chaos game representation (CGR) was proposed in 1990 [38]. The specific steps are as follows: each of the four bases corresponds to a vertex of a unit square. Starting from the center of the square, a line is drawn connecting the center to the vertex corresponding to the first nucleotide, and the midpoint of this line segment represents the first nucleotide. The second nucleotide is represented by the midpoint of the line between the point of the previous nucleotide and the corresponding vertex of the second nucleotide, and this process is repeated (Figure 4a). CGR significantly reduces the degeneration problem while exhibiting a “compactness feature”, enabling the representation of longer DNA sequences within a limited space. The graphical representation produced by CGR generates a DNA fractal, revealing the inherent structure and characteristics of the DNA sequence. For instance, if the DNA sequence contains repeated nucleotides or nucleotide fragments, or if certain nucleotides are missing, the points within the square region will become denser in some areas and sparser in others. The features extracted from the CGR highlight the overall pattern of the DNA sequence. If no significant pattern exists within the sequence, the points in the CGR will be randomly distributed; however, if a pattern is present, it will exhibit a fractal structure.

FCGR (Frequency Chaos Game Representation) is a digital frequency matrix derived from the CGR. The k-th order FCGR for a DNA sequence is represented as a $2^{k}\times 2^{k}$ matrix [39]. Each matrix element in FCGR corresponds to the number of points in each grid of the CGR (which represents how many times a particular *k*-length nucleotide appears), i.e., the frequency of k-mers (subsequences of length *k*, of which there are $2^{k}$ possible types). FCGR provides a matrix of the frequencies of k-mers extracted from the DNA sequence. This matrix can be conveniently processed by neural networks, opening up various applications and offering a new perspective on biological sequence analysis [40].

The main concept behind the WormBin visualization model is to convert DNA sequences into binary codes and label them on the worm curve [41,42] (Figure 4b). The resulting curve is sufficiently dense to represent long sequences within a limited space. Randic et al. used this graphical representation to compare the DNA sequences of the first exon of the *human β-globin* gene and the *gorilla β-globin* gene. Zhang et al., building on WornBin, proposed two new methods for sequence similarity analysis [43]. However, it is difficult for observers to identify which base is represented by each point on the curve. The main concept behind the WormStep is to indicate the four bases by moving a specific number of steps along the worm curve, with the corresponding step counts as follows: A→1 step, C→2 step, G→3 step, and T→4 step [44] (Figure 4b). WormStep allows for easier determination of the base composition of a DNA sequence by calculating the distance between two points on the curve. Zhang used WornStep to perform similarity analysis and construct a phylogenetic tree based on the complete coding DNA sequences of the *β-globin* genes from ten species.

Furthermore, Color5 improved the four-color model and has the same density advantage as WormBin [45,46]. This model’s distinctive feature is the use of different colors to represent the bases, with color assignments as follows: A, red; T, green; G, blue; and C, yellow. It is designed as a compact two-dimensional structure resembling a vortex structure. Adjacent blocks representing the same base are painted the same color, and the edges between them are removed. Blocks of the same color can be labeled sequentially (Figure 4c). Color5 can be converted into a digital matrix, which can then be used to extract two types of numerical features: a 24-dimensional vector representing the matrix eigenvalues and a 96k-dimensional vector for the checksum. Randic et al. used these two features for similarity analysis. The four-base related curve (F-B curve) is based on trigonometric functions along with the corresponding single-base related curves (A-, G-, T-, and C-related curves) [47]. It uses sine and tangent functions to represent the change process of the four bases (Figure 4d). Xie et al., based on this curve, constructed an 8-component feature vector to compare the similarity of DNA sequences between different species. They compared the similarity of the first exon coding sequences of the *β-globin* gene across 11 species, the cDNA sequences of the *β-globin* gene from 8 species, and the entire mitochondrial genomes of 18 species of placental mammals, using the normalized geometric center of the proposed curve as a reference.

In addition, some visualization models are based on the construction of a unit circle. For example, the B-curve maps each base to a unit circle by constructing mapping [48] (Figure 4e). Wang et al., based on the B-curve, studied the first exon sequences of the 13 globin genes from 11 different species. They constructed a similarity matrix and built a phylogenetic tree, performing a similarity analysis on these sequences. They further applied this method to the mitochondrial gene sequences of 45 species, and the results were largely consistent with biological evolutionary relationships. The DUC curve also maps four types of bases on a unit circle [49]. The DUC curve is constructed by mapping the four nucleotides in a cyclic order onto the unit circle. Since all points in this graphical representation of the DNA sequence are arranged along the circumference of the unit circle, we refer to it as the “DUC Curve”. However, when dealing with long sequences, the DUC curve becomes overly dense, leading to a compromised visualization effect (Figure 4f). The DUC curve can directly detect nucleotide, dinucleotide composition, and microsatellite structures within DNA sequences. Additionally, it can be used for DNA sequence alignment. Li et al. used the geometric center vector of the DUC curve as a sequence descriptor and, with a lower computational cost, established reasonable phylogenetic relationships for the first exons of the *β-globin* gene from 11 species, the *TP53* gene from 27 species, and the *TP53* gene from 24 strains of H1N1 influenza virus.

It can also combine adjacent nucleotides with a unit circle to position the 16 pairs of adjacent nucleotides on the unit circle, deriving the corresponding mapping angles. Based on these mappings, the respective coordinates for these 16 dinucleotides are obtained [50,51] (Figure 4g). This method allows for visual inspection of dinucleotide-based data and can easily compute sequence invariants. It also avoids the complexity of computational sequence alignments, particularly for long, fully encoded sequences. Liu et al. demonstrated this by analyzing the similarities and differences in the complete coding sequences of the *β-globin* gene across 11 different species.

## 3. Three-Dimensional Visualization of DNA Sequences

### 3.1. Regular Tetrahedral Model

**Figure 5:**
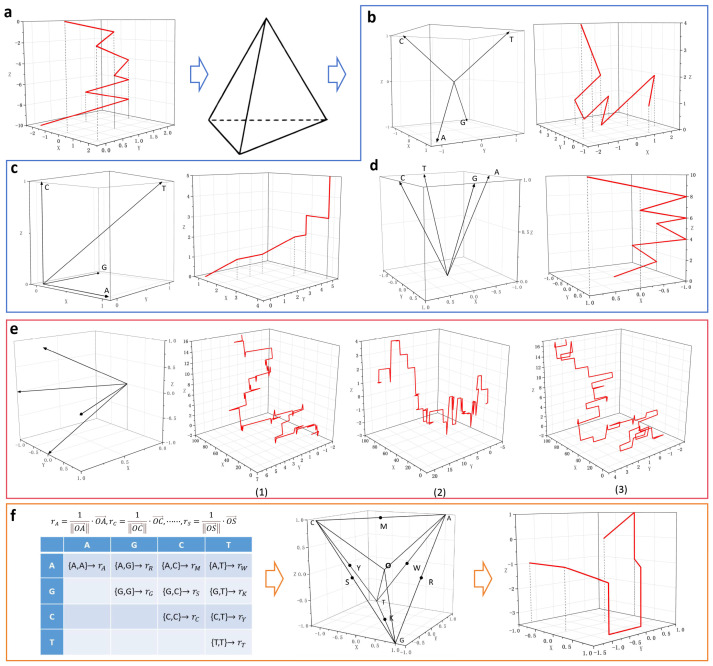
3D visualization methods based on the tetrahedral model and the corresponding graphical representations (the sequence used is the first exon of the *human* and *goat β-globin* genes). (**a**) H-curve. (**b**) Method of Randic et al. [53] (**c**) Method of Wang et al. [54] (**d**) Method of Wąż et al. [55] (**e**) The method combines three classifications of nucleotides, RY (1), MK (2), and SW (3) curves. (**f**) The method combines the multiset and 2-combinations of bases, vector assignment table, and vector design for regular tetrahedra.

The G- and H-curves were proposed by Hamori et al. [52] as some of the earliest graphical representation methods for quantitatively describing DNA sequences. The G-curve is generated in a virtual 5-dimensional space, where the first four components of the coordinates correspond to the four nucleotide bases, and the final component represents the positional features of the nucleotides in the sequence. Since the G-curve exists in a 5-dimensional abstract space, it is difficult to intuitively understand or visualize. The H-curve shown in Figure 5a overcomes the drawbacks of the G-curve by presenting the DNA sequence in a three-dimensional graphical representation, facilitating easier observation. The H-curve first defines a function g(*z*), where *z* represents the position of a nucleotide in the DNA sequence. If the base at position *z* is A, then g(*z*) = *i* + *j* − *k*; if the base is T, then g(*z*) = *i* − *j* − *k*; if the base is C, then g(z) = −*i* − *j* − *k*; and if the base is G, then g(z) = −*i* + *j* − *k*. Here, *i*, *j*, and *k* are the unit vectors along the coordinate axes in a three-dimensional space. Next, a function is defined $H_{i,j}(z)\equiv \sum_{1}^{z}g\left(z\right)$. When H(*z*) spans from position *i* to position *j* in the DNA sequence, it generates a series of points in space. By connecting the adjacent points sequentially, the H-curve is obtained. However, the H-curve might deviate from the Z-axis, which could hinder feature analysis. Hamori et al. used the H-curve to analyze the DNA sequence of the antibiotic M13 and identified the mutated regions within the sequence. They observed that the starting points of all genes were guided by short, purine-rich sequences, which provided a sensitive signal for intuitively identifying genes. Other viral genes, such as those of the Human Immunodeficiency Virus (HIV) and Epstein-Barr Virus (EBV), have also been studied using the H-curve.

Multiple representations may arise from many two-dimensional graphical representations when the vectors corresponding to the four nucleotides are not equivalent. To eliminate this ambiguity, multiple interpretations can be avoided if four equivalent vectors are present. Considering that the vectors from the center of a regular tetrahedron to each vertex are equivalent, a regular tetrahedron can be used. This approach maps the four bases to the four vertices of a regular tetrahedron, as shown in Figure 5. In this analysis, the center of a regular tetrahedron is taken as the origin of a Cartesian (*x*, *y*, *z*) coordinate system, and the coordinates of the four vertices are assigned to the four nucleotide bases.

The initial method was proposed by Randic et al., but it can encounter degeneracy issues [53]. Randic et al. used this method to introduce the first distance-matrix-based graph theory approach, treating it as a quantitative feature for comparing sequence similarity. The first method based on this model showed degradation, but a series of non-degenerate methods have been proposed since then [54,55,56] (Figure 5b–d). These methods essentially outline a way to quantify similarity by constructing a three-component vector (with its components representing the geometric center) and a four-component vector composed of the graphical radius associated with the DNA curve. The practicality of these methods was demonstrated through experiments comparing the similarity and differences between the first exon coding sequences of the *β-globin* gene from 11 species. These methods can also combine three nucleotide classifications [57] (Figure 5e), leading to a 3D graphical representation proposed by Xie et al., known as the RY curve, MK curve, and SW curve. The two-dimensional projections of these curves in the plane have clear biological significance. Based on these curves, Xie et al. constructed a 12-component feature vector to compare the similarity between the first exon coding sequences of the *β-globin* gene from 11 species and validated the similarity of the cDNA sequences of the *β-globin* gene from 8 species.

The set of all distinct dinucleotide combinations obtained from the set {A, C, G, T} consists of 6 pairs. Li et al. replaced the set {A, C, G, T} with a multiset [∞∙A, ∞∙G, ∞∙C, ∞∙T], allowing for repetition, which results in 10 possible 2-combinations [58]. Then, the four nucleotides—A, C, G, and T—are mapped to the vertices of a regular tetrahedron. Ten unit vectors are obtained by denoting the midpoints of the segments AC, GT, AG, CT, AT, and CG as M, K, R, Y, W, and S, respectively. By associating these 10 nucleotide 2-combinations with the corresponding 10 unit vectors, and using a sliding window approach to read dinucleotides in the DNA sequence, the corresponding vectors can be shifted to convert the sequence into a three-dimensional graphical representation (Figure 5f). Li et al. used this method to construct cell-based descriptor vectors for the numerical characterization of DNA sequences. They performed phylogenetic analysis on four datasets: the *globin* genes from 18 species, the *mitochondrial cytochrome oxidase (COI)* genes from 9 species of butterflies, the S segment of 32 hantaviruses (HVs), and the complete mitochondrial genomes of 70 species.

### 3.2. Models Extending Upward Along the z-Axis

**Figure 6:**
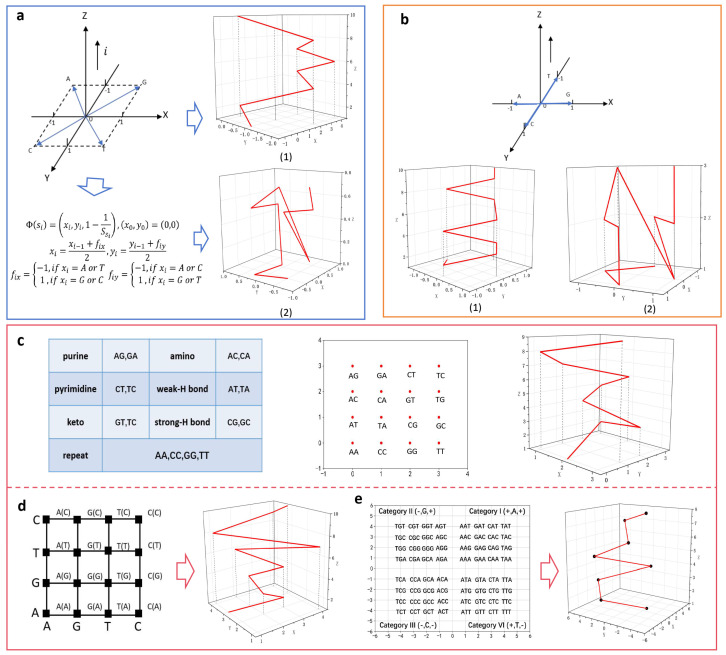
3D visualization methods of the resulting curves extending along the Z axis and the corresponding graphical representations (the sequence used is ATGGTGCACC). (**a**) The method of Wu et al. [59] (1) integrates the coordinates mapping of bases with CGR (2). (**b**) The method counts the number of bases (1) and the number of times the current base is repeated (2). (**c**) The method allocates 16 types of dinucleotides to the matrix, the classification table of dinucleotides, and the coordinate assignment. (**d**) The method combines spectral models and two-dimensional spectral graphs arranged in AGTC order. (**e**) The method of Yu et al. [60] and the distribution of triplets on the plane of coordinates.

Three-dimensional visualization methods that extend along the z-axis fix the values of the x and y components of their base vectors, with the z component increasing from 0 as the number of bases increases; thus, the resulting curve is extended up to the z-axis. This method effectively prevents degeneracy and information loss, as shown in Figure 6.

**Figure 7:**
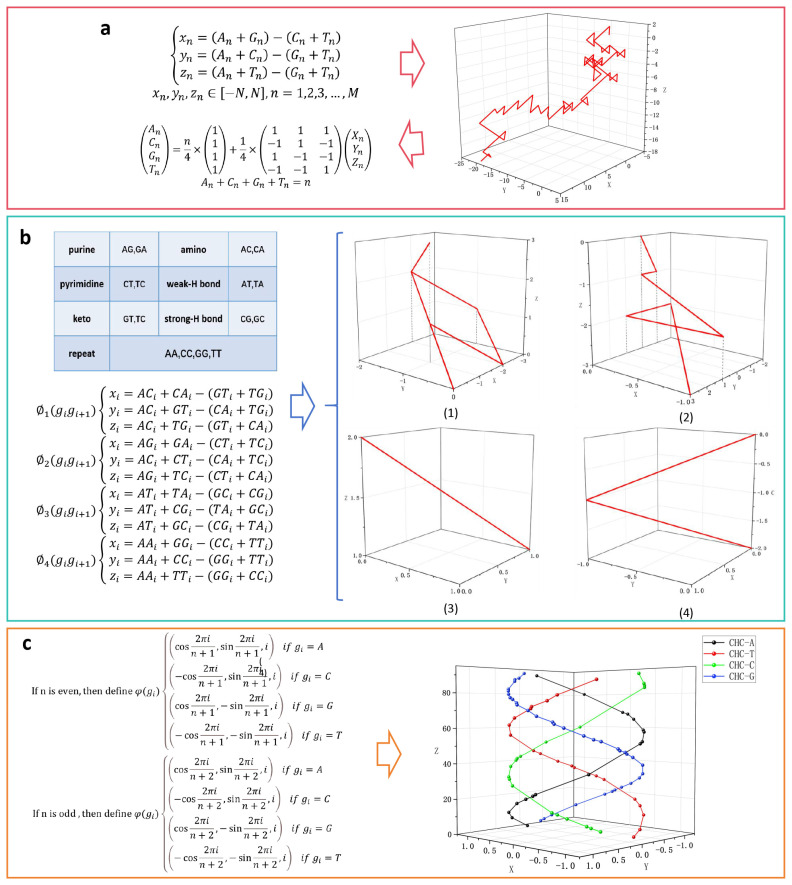
3D visualization method based on the special model and the corresponding graphical representations (the sequence used is the first exon of the *human* β-globin genes). (**a**) Z-curve. (**b**) The method combines the physicochemical properties of adjacent nucleotides. (**c**) Circular helix curve (CHC).

This type of method was first proposed by Wu et al., where the four nucleotides are assigned to the vertices of a unit square with A at (−1, −1), T at (1, 1), C at (−1, 1), and G at (1, −1). The Z-axis represents the count of each nucleotide. By sequentially connecting the resulting coordinate points, a graphical representation of the DNA sequence is obtained. Wu et al. applied this curve to analyze HIV and RNA viruses, achieving significant results. An improved method maintains the same vector design, integrating the coordinates mapping of bases with CGR (Chaos Game Representation) [59] (Figure 6a). Another method involves changing the design of the vector. In addition to counting the number of bases, the z-component value can also represent the number of times the current base is repeated in the sequence [61,62,63] (Figure 7b). Liao et al. demonstrated the practicality of this approach by examining similarities and differences among the first exon coding sequences of the *β-globin* genes across various species.

Similar models have been constructed using the distribution of nucleotide combination on a coordinate plane. The PN curve, where PN stands for “pair of nucleotides”, allows for the definition of a matrix distance (MD) between matrices derived from different PN curves. Qi et al. used the MD measure to characterize and compare the complete coding sequence regions of the *β-globin* gene in seven different species. For instance, by allocating 16 types of dinucleotides to the matrix according to their physicochemical properties [64,65] (Figure 6c). Another method combines spectral models by considering each individual base and the next connected base [48]. The horizontal line on the x-axis corresponds to the current base type, whereas the horizontal line on the y-axis corresponds to the connected base. The intersection points of the two horizontal lines represent the x- and y-components of the current base (Figure 6d). This method considers both the structural aspects of DNA sequences and the connections between nucleotides, which allows the graphical representation to capture more comprehensive DNA sequence information. Consequently, this leads to a more rational measurement of sequence similarity. Li et al. applied this approach to analyze the similarity of the first exon in the *β-globin* gene across 11 species, achieving favorable results.

The TN (Trinucleotides) curve is based on nucleotide triplets, which considers the distribution of nucleotide triplets in the sequence [60,66] (Figure 6e). Since the four nucleotides (A, G, T, C) can form 64 unique trinucleotides, and the second base in each trinucleotide is often associated with the hydrophobic/hydrophilic properties of the corresponding amino acids, these 64 trinucleotides can be classified into four groups based on this characteristic. Using the TN curve, Yu et al. developed two straightforward methods to analyze similarity, dissimilarity, and conserved gene regions among coding sequences of different species. These methods, which employ two vectors composed of 64 and 6 components respectively, are efficient, are easy to execute, and have low computational costs.

### 3.3. Other Special Models

A three-dimensional visualization method based on a special model is shown in Figure 7.

Some methods make full use of the frequency and positional information of the bases in a sequence when obtaining the three-dimensional coordinates for graphical representation. These methods exhibit strong numerical characteristics and involve intricate mathematical expressions. For example, the Z-curve calculates the counts of A, C, G, and T bases from the first base to the current base in a DNA sequence of length M [67,68]. The point coordinates can be determined using these four positive integers. The connection of these points yields the Z-curve (Figure 7a). The Z-curve provides the coordinates of each point and can be used to reconstruct a DNA sequence using a mathematical model.

The Z-curve not only encapsulates the biological characteristics of DNA sequences but also provides a unique representation of nucleotide sequences. However, if all nucleotide frequencies are identical, the transformed spatial curve may exhibit loops. The three components of the Z-curve have distinct biological significance and carry strong mathematical implications. The symmetry, periodicity, local motifs, and global feathering of nucleotide distribution are reflected in the Z-curve’s intricate folding structure. The Z-curve can be further smoothed using B-spline functions of varying orders, expanding the potential for using geometric methods to visualize and analyze DNA sequences. It has broad applications in comparative genomics, gene prediction, base composition calculations, as well as identifying and predicting replication origins and termini in bacterial and archaeal genomes.

Therefore, another method combines the physicochemical properties of adjacent nucleotides and presents DNA sequences as four graphical representations [69] (Figure 7b). Cao et al. utilized this graphical representation along with a covariance matrix to compare DNA sequences and proposed a new similarity measurement approach. The Circular Helix-like Curve (CHC) consists of CHC-A, CHC-C, CHC-G, and CHC-T, which correspond to the arrangement and distribution of the four bases in the sequence [70]. The curve of base A in a sequence is similar to a cylindrical helix, known as the circular helix curve of base A (CHC-A). Similarly, CHC-C, CHC-G, and CHC-T can be generated (Figure 7c). The CHC curve captures the positional and distributional patterns of bases within a DNA sequence, making it useful for identifying similarities and differences among multiple DNA sequences. Li et al. extracted a 12-dimensional vector from the CHC as a numerical representation and used it to analyze the phylogenetic relationships of 11 species, 74 types of ribosomal RNA, 48 strains of hepatitis E virus, and 18 eutherian mammals.

## 4. Knowledge Graphs for Biological Sequence Visualization

### 4.1. Background

A knowledge graph integrates theories and methods from disciplines such as mathematics, graphics, and information sciences. It uses visual means to showcase the structure, patterns, and distribution of knowledge. Over the past few decades, the visualization of biological sequences has developed rapidly, leading to the implementation of numerous visualization methods. These methods are scattered across research databases in various forms such as research papers and reports. Retrieving the desired visualization methods and acquiring essential details, such as basic ideas, advantages, and disadvantages, can be cumbersome. Consequently, there is a deficiency in tools that can collect, organize, and logically establish the relationships among these visualization methods. Knowledge graphs are an effective method for organizing and managing extensive datasets and can extract structured knowledge from vast sets of data, significantly reduce human labor, and provide a foundation for the effective utilization of relevant knowledge [71,72,73].

### 4.2. Theory and Model of Construction

**Figure 8:**
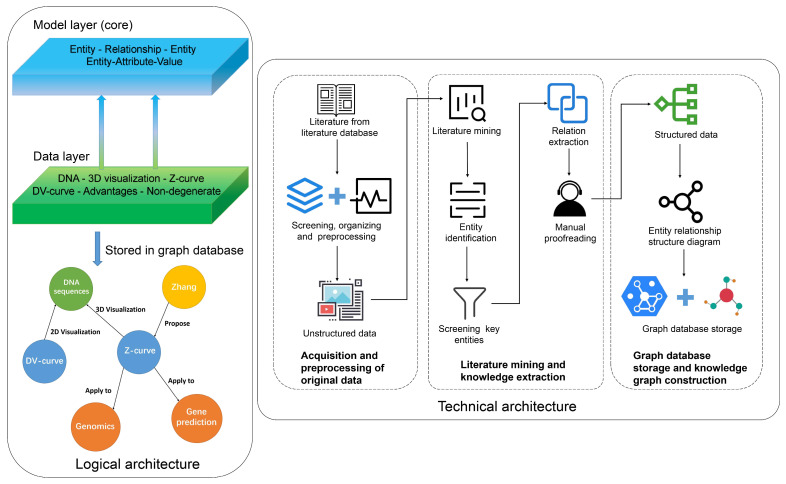
Overall architecture of the knowledge graph for biological sequences visualization.

The construction of a knowledge graph for biological sequence visualization is based on literature mining. Literature mining can be used to extract relationships between biological sequences and visualization methods from research papers to create a knowledge repository for this subject. The process begins by collecting original data from a research database and using literature-mining tools to construct a relevant research dataset. Next, knowledge extraction techniques are applied to gather pertinent information. Lastly, the information is stored in graphical format in the graph database after manual review [74,75,76]. Figure 8 illustrates the overall architecture.

Knowledge graphs can be classified into logical and technical architectures. The logical architecture consists of schema and data layers. The schema layer is the core of the knowledge graph and is located above the data layer. It primarily defines the data structure of knowledge, including the hierarchy and relationships of knowledge classes, such as entities, relationships, and attributes. The schema layer defines a specific form of knowledge within the data layer. The data layer is mainly composed of a set of facts presented in the form of triplets. As demonstrated in Figure 8, we identified triples such as “Z-curve—3D visualization—DNA sequence” and “DV-curve—2D visualization—DNA sequence”. Knowledge is stored in units of facts, and graph databases are used as storage media. All data stored in the graph database constitute a huge entity knowledge network and form a knowledge graph. Our ultimate goal is to construct an entity knowledge graph for biological sequence visualization methods. The architecture comprises three main components: the acquisition and preprocessing of original data, literature mining and knowledge extraction, and graph database storage and knowledge graph construction [77,78,79].

To ensure the reliability of the data, they are gathered from widely recognized research databases such as PubMed and Web of Science. The abstracts usually comprise research objectives, methods, results, and conclusions, thereby covering the main content of the articles. Therefore, keywords like “curve”, “DNA”, “protein”, “graphical representation”, and similar terms can be used to initially retrieve the relevant literature. Subsequently, the literature retrieved is filtered and sorted based on the abstracts, and the eligible literature is exported as a data source. Lastly, natural language processing techniques are used to preprocess the literature. Preprocessing involves removing irrelevant data, such as author details and publisher information, while retaining crucial information from the literature. This provides the necessary unstructured data for subsequent steps [80,81].

To process a large amount of unstructured literature data containing natural language descriptions, we employ literature mining techniques [82,83,84]. However, given the specific domain of biological sequence visualization, which involves numerous domain-specific entity names, automatic text mining tools would inevitably miss a significant number of entities. Therefore, we initially create a training set through manual annotation and utilize pre-trained models and deep learning methods like FabNER to perform named entity recognition [85,86,87,88]. This process allows us to filter out irrelevant entities and identify key entities related to visualization methods.

Subsequently, for relation extraction, we follow a similar approach by manually annotating the relationships between visualization methods and other entities, such as authors, application domains, and graphical representations. After training a model on this annotated data, we apply it to extract relationships from other texts in the literature. The extracted relationships are then output in the form of triples: {‘subject:’, ‘relation:’, ‘object:’}. Since the extraction results may have multiple possibilities, further manual filtering and verification are required to obtain structured data [89,90].

Finally, we focus on the storage and construction of the knowledge graph. First, entity types are determined through a comprehensive analysis of structured data, and an entity relationship structure diagram is constructed to establish the types of relationships between entities, and between entities and attributes. When establishing relationships, the structured data obtained are read by data processing tools such as Python and R. Based on the relationship structure obtained, data can be stored as parent–child nodes. The defined entity nodes and relationships are stored in a graph database. As entities and their relationships are built using triples, two types of triple relationships must be established: entity–relation–entity and entity–attribute value. Eventually, a knowledge graph can be constructed [91,92].

The knowledge graph constructed not only enables the rapid retrieval of the desired visualization methods and their related information, but also facilitates the discovery of other relevant methods. This allows us to discern the differences among various methods, providing a knowledge base to support research in the field of biological sequence visualization. The process of building this knowledge graph can be viewed as a comprehensive review of the research landscape in biological sequence visualization, offering a fresh analytical perspective and showcasing the research trends. This provides a reference for the application of intelligent search engines, knowledge base completion, knowledge question-answering systems, and other applications in biological sequence visualization.

## 5. Machine Learning Methods in Biological Sequence Visualization

### 5.1. Overview

**Figure 9:**
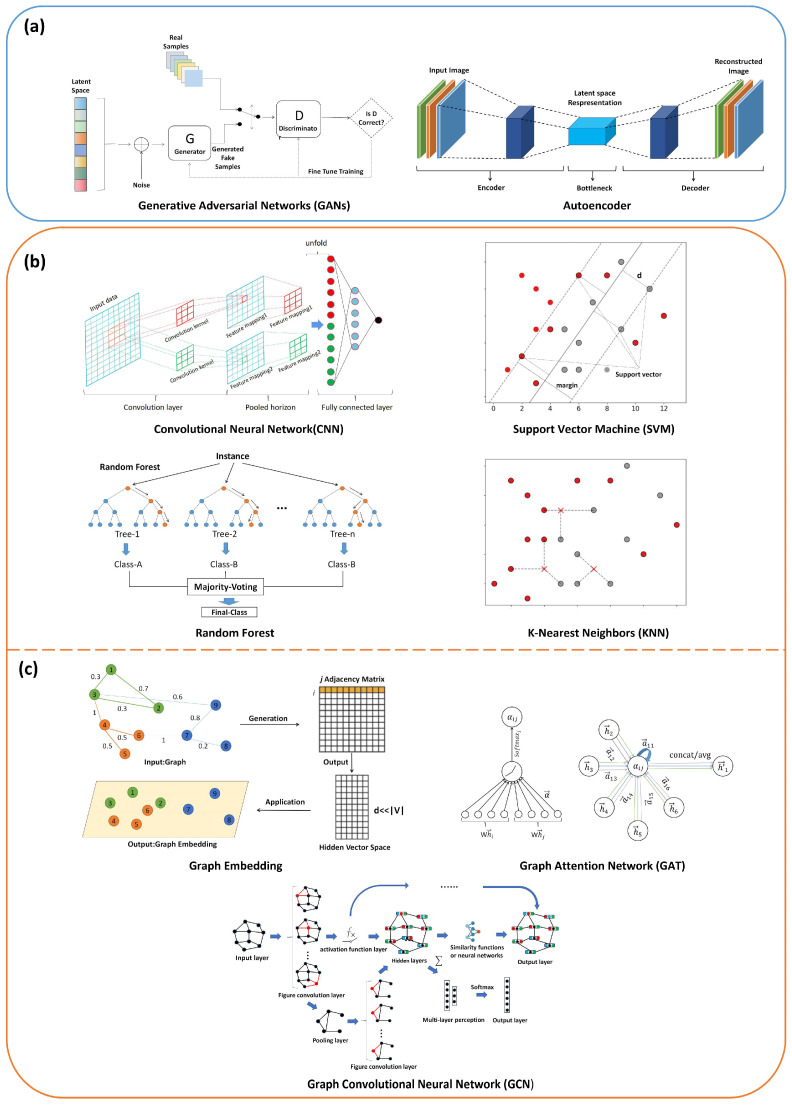
Summary of the models and structures of machine learning methods. (**a**) Machine learning methods for generating graphical representations of biological sequences. (**b**) Machine learning methods for processing graphical representations of biological sequences. (**c**) Machine learning methods for processing graph representations of biological sequences.

The application of machine learning methods to biological sequence visualization can be approached from two perspectives. The first approach involves studying how machine learning methods can be used to generate graphical representations of biological sequences. The second approach involves using machine learning methods to study the graphical representation generated, including feature extraction and classification prediction. Considering these two aspects, machine learning methods that can transform sequence data into graphical representations, which can effectively handle images and graph structure data, hold significant potential in terms of research value [93,94,95,96,97], as illustrated in Figure 9.

Relatively few machine learning methods can generate graphical representations based on sequence data. These fall into two main categories: generative adversarial networks (GANs) and autoencoders, as illustrated in Figure 9a. GANs are adversarial models consisting of a generator and discriminator. By training the generator to create images from sequence data as input and then adversarially training it against real images, the generator can learn to produce images with similar features [98,99,100,101]. An autoencoder is an unsupervised learning method. It consists of an encoder and a decoder. The encoder maps the graphical representation of the biological sequences to a low-dimensional space, which is reconstructed using a decoder. Autoencoders can learn meaningful feature representations of biological sequences [102,103].

Machine learning algorithms commonly used for graphical representation processing include classical convolutional neural networks (CNNs), support vector machines (SVMs), random forests (RFs), and K-nearest neighbor (K-NN) algorithms, as illustrated in Figure 9b. CNNs are widely used in tasks such as computer vision, image classification, and recognition. The SVM is a supervised learning algorithm that discriminates and classifies image data by finding an optimal hyperplane in the feature space [104,105]. RF is an ensemble learning algorithm composed of multiple decision trees. It makes predictions and analyzes images based on collective decision-making [106,107]. The K-NN is a common nonparametric learning algorithm that compares unknown images with the nearest training samples based on similarity metrics, thereby facilitating tasks such as classification and retrieval [108].

The application of graph theory for the visualization of biological sequences is rapidly growing. Graph-embedding methods, graph convolutional networks, and graph attention networks can be used to handle the resulting graph structure data, as illustrated in Figure 9c. Graph convolutional networks (GCNs) are a class of neural network models designed to perform convolution operations directly on graph data (e.g., graphs composed of nodes and edges). Unlike traditional convolutional neural networks (CNNs), which perform convolutions on regular grid data (such as images), GCNs define convolution operations based on the adjacency relationships of the graph structure, enabling the processing of node features and edge weights in graph data [109,110,111,112]. A graph attention network (GAT) is a type of graph neural network based on attention mechanisms. It learns the importance weights between each node and its neighboring nodes [113,114]. The details of the graph-embedding method are discussed below. The following sections delve into two potential applications of machine learning methods in biological sequence visualization. Firstly, we explore the use of graph embedding techniques to represent biological sequence graph data as vectors, enabling further application in machine learning and deep learning tasks. Secondly, we discuss the application of convolutional neural networks (CNNs) to learn from the graphical representations of biological sequences for specific biological sequence prediction tasks.

### 5.2. Specific Application Direction

#### 5.2.1. Vector Representation of Sequence Graphs Based on Graph Embedding Methods

**Figure 10:**
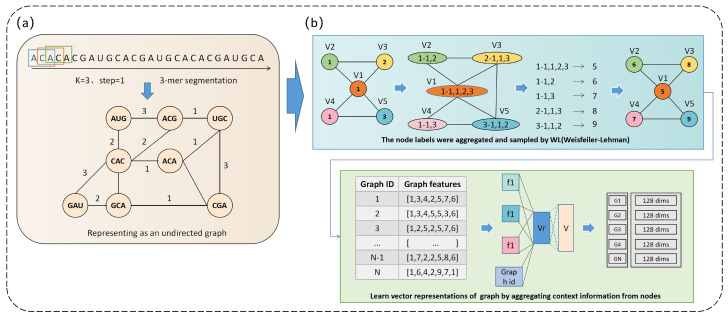
Vector representation of sequence graphs based on graph embedding methods. (**a**) Undirected graph representation of RNA sequences. (**b**) Graph vector representation based on Graph2vec.

This section primarily focuses on a potential application involving the use of graph embedding techniques to map biological sequence graph data into a low-dimensional space. By representing nodes and edges in the graph as vectors, we can apply more machine learning- or deep learning-based biological sequence prediction tasks [37]. This section provides a brief overview of sequence graph construction, using the undirected graph representation of RNA sequences as an example. First, adjacent k-mers within a sequence are connected and stored in a graph data structure. Specifically, the adjacent 3-mers are divided and linked together, with the edge weights corresponding to the occurrence count of the adjacent 3-mers, as illustrated in Figure 10a. After converting the RNA sequences into graphs, graph embedding methods are used to represent them as vectors. Graph embedding is a technique that maps graph data to a lower-dimensional vector space. It effectively translates graph nodes and edges into vectors for applications in deep learning tasks [115,116,117,118].

For example, Graph2vec can represent all graphs as vectors. This is accomplished by aggregating and ordering the label values of adjacent nodes in the graph multiple times, constructing graph node label sequences, and incorporating context information and graph IDs to learn graph representations [119]. Graph2vec uses a shallow neural network based on a continuous bag of words (CBOWs). It takes the context information of node labels as input and output and is trained to obtain the vector representation of the graph [120,121] (Figure 10b). The Weisfeiler–Lehman (WL) method is used to aggregate the labels of adjacent nodes in the graph, generating a sequence containing all the node labels. Subsequently, the CBOW model learns the vector representation of each graph based on the information from the node label sequence of the graph [122,123]. Once biological sequence graphs are converted into vector representations, numerous applications become possible. For example, graph embeddings of RNA sequences can be used to construct gene regulatory networks, identifying the crucial roles of key transcription factors, miRNAs, long non-coding RNAs, and other elements in the regulatory process. Additionally, graph embeddings can be applied to predict RNA–protein interactions, aiding in the identification of proteins that bind to RNA molecules and revealing their synergistic functions within cells [124].

#### 5.2.2. Processing of Biological Sequence Graphical Representations Based on CNN

**Figure 11:**
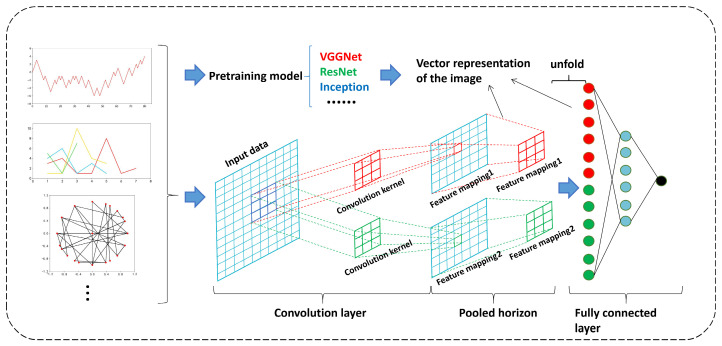
Classification prediction and feature vector extraction of graphical representation based on CNN.

This section investigates the application of convolutional neural networks to learn from the graphical representations of biological sequences for specific biological prediction tasks. CNNs have demonstrated exceptional performance in feature extraction and image recognition. Therefore, utilizing CNNs to extract features from graphical representations of biological sequences and applying them to biological sequence-based prediction tasks is highly meaningful. A CNN is a deep learning model suitable for data processing with correlated features. CNNs operate using convolutional kernels as their basic learning units and scan along the horizontal and vertical directions of the input data to automatically extract relevant features. The extraction process is recorded using the weights of the convolutional kernels. By using the error between the input and output data, CNNs update the learning parameters of their kernels through backpropagation, dynamically learning the features of the input data and updating the network parameters [125,126,127,128]. Therefore, CNNs can be used to investigate graphical representations of biological sequences. Moreover, it is possible to input graphical representations into pretrained CNN models, with commonly used pretrained models, including VGGNet, ResNet, and Inception. Figure 11 illustrates the process of visualization, classification prediction, and feature vector extraction based on the CNN.

In the fully connected layer and the global average pooling layer, the feature vectors from the second-to-last layer can be used as vector representations for images. These vectors capture high-level features within the image and can be used for subsequent tasks, such as analysis, classification, and clustering, using machine learning or deep learning methods [128,129]. For instance, Zhou et al. employed chaos game representation and convolutional spiking neural networks for gene essentiality prediction. By using convolutional spiking neural networks (SNNs) with reward-modulated spike-timing-dependent plasticity (R-STDP) learning rules to learn from the frequency matrix chaos game representation (FCGR) images of essential and non-essential genes, they achieved accurate within- and cross-organism essential gene prediction and demonstrated a simple and reliable method for predicting essential genes [39]. This spiking neural network can make better predictions of essential genes by directly extracting gene features from the FCGR images of essential and non-essential genes.

## 6. Summary and Prospects

First, this study focuses on the 2D and 3D visualization methods for DNA sequences and provides a comprehensive categorization of most of these methods. In addition, this study elaborates on the theoretical framework, methods, and models for constructing a knowledge graph for biological sequence visualization. Furthermore, it introduces machine learning methods applicable to biological sequence visualization, with specific discussions on the applications of graph-embedding methods and CNNs. A notable feature of this study is not only an extensive summary of DNA sequence visualization methods, but also an analysis of the commonalities between different methods and their introduction in a classification. Current biological sequence visualization methods have solved the problems of degradation and information loss. However, there are still problems such as complex calculation processes, and difficulties in representing all information contained in the sequence, accurately restoring the effective information of the sequence, and observing long sequences. Therefore, additional visualization models that can overcome these shortcomings must be designed.

The knowledge graph construction method proposed in this study for visualizing biological sequences offers novel technical references and analytical perspectives. This approach provides insights that can benefit various disciplines including biology, medicine, and computer science, spanning areas such as computational biology, bioinformatics, and image processing. In addition, it serves as a reference for applications such as intelligent search engines for biological sequence visualization, knowledge base completion, and knowledge question-answering systems. In future research on biological sequence visualization, several avenues warrant in-depth exploration. First, finding outstanding visualization methods. Second, analyzing biological sequence visualization methods based on knowledge graph. Third, the integration of advanced machine learning methods for generating and analyzing graphical representations of biological sequences to achieve applications such as classification prediction and feature extraction. Lastly, extending the applications of biological sequence visualization. Owing to space constraints, not all biological visualization methods are exhaustively discussed in this study, which may have resulted in the omission of some visualization methods. Furthermore, the construction of knowledge graphs and interpretation of machine learning methods are based on existing knowledge and experience. In future studies, we will continue to focus on the visualization of biological sequences and delve deeper into the application of knowledge graphs and machine learning in this domain.

## Data Availability

Not applicable.
